# Association between family functioning and psychotic transition in ultra-high risk adolescents and young adults

**DOI:** 10.3389/fpsyt.2023.1177311

**Published:** 2023-06-21

**Authors:** Vladimir Adrien, Justine Liewig, Thomas Diot, Florian Ferreri, Stephane Mouchabac, Caroline Dubertret, Julie Bourgin

**Affiliations:** ^1^AP-HP, Department of Psychiatry, Saint-Antoine Hospital, Sorbonne Université, Paris, France; ^2^Infrastructure for Clinical Research in Neuroscience (iCRIN), Paris Brain Institute, Sorbonne Université, INSERM, CNRS, Paris, France; ^3^Department of Child and Adolescent Psychiatry, Nord-Essonne Hospital, Bures-sur-Yvette, France; ^4^AP-HP, Department of Psychiatry, Louis Mourier Hospital, Université Paris Cité, Faculté de Médecine, Colombes, France

**Keywords:** psychotic transition, family functioning, environment, adolescent, FAD-60

## Abstract

**Background:**

Psychotic transition (PT) is a crucial stage in schizophrenia. The Comprehensive Assessment of At-Risk Mental States (CAARMS) scale can be used to identify individuals at ultra-high risk (UHR) for psychosis and to evaluate their risk of PT. Many environmental and genetic factors have been identified as contributing to the development and decompensation of schizophrenia. This study aimed to determine if the quality of family functioning is associated with PT risk in UHR individuals aged between 11 and 25 years after 1 year of follow-up.

**Methods:**

From January to November 2017, 45 patients aged 12 to 25 consulting for psychiatric reasons were included. Twenty-six were classified as UHR of PT at the CAARMS. Family functioning was assessed by the Family Assessment Device—Global Functioning (FAD-GF). Thirty-seven of these patients (30% men, mean age 16 ± 2.5) were reassessed at 8–14 months of recruitment. Survival analysis was used to examine the impact of family functioning on PT risk.

**Results:**

A total of 40% of UHR patients were classified as psychotic at reassessment. Survival analysis showed that better family functioning is a significant protective factor for PT in this population.

**Discussion:**

This result suggests that the global family functioning has an impact at 1 year on the risk of PT in the population of adolescents and young adults who consult the hospital for psychiatric reasons. A family intervention may be effective in reducing PT risk in this population and should be considered as a potential therapeutic option.

## 1. Introduction

Schizophrenia is a severe chronic psychotic disorder affecting 1% of the population worldwide (1, 2). The first decompensation, called psychotic transition (PT), usually occurs between late adolescence and early adulthood. The prodromal phase of psychosis, characterized by the presence of attenuated or time-limited psychotic symptoms and a decline in psychosocial functioning, can be used to identify young individuals at ultra-high risk (UHR) for psychosis (3). UHR patients most often have some functional, cognitive, and social limitations with an impairment that leads them to seek help. Early identification and intervention during the prodromal phase can improve outcomes (4) and potentially prevent the onset of full-blown psychosis. The use of the Comprehensive Assessment of At-Risk Mental States scale (CAARMS) allows for the clinical assessment of patients (5–7).

Multiple studies in the literature have indicated that schizophrenia is a multifactorial disease, with both genetic and environmental risk factors (8) contributing to its development. Genetic vulnerability factors have been identified, as evidenced by the high concordance rate of 50% in monozygotic twins (9). However, this concordance rate also highlights the role of environmental factors in the development of the disease. The interplay between these genetic and environmental factors is known as gene–environment interaction.

Many environmental factors (8, 10) have been and are still studied, like perinatal complications, trauma to the central nervous system, substance use disorders, migration, or psychotrauma. Regarding pathophysiological hypotheses, many have been proposed for the genesis of schizophrenia: the dopaminergic hypothesis (11), the glutamatergic hypothesis (12), or most recently membrane hypothesis (13). All of them are interrelated and linked to current research on immunity, inflammation, hormonal system, intestinal microbiota (14), microglia (15), and, in general, to environmental factors that influence these various biological factors.

If family environment impact has been well-assessed for the prognosis of many other psychiatric conditions, like depression (16–18) or eating disorder (19–21), few were conducted for psychotic conditions (22) and only transversal assessments were made. Hur et al. (23) showed that a high socioeconomic level (SEL) contributes to a significant improvement in the symptoms of UHR subjects in the 1st year of follow-up, but no significant clinical difference was found after 2 years of follow-up. PT rate was not different according to the SEL. Another study showed that the behavior of the family toward the UHR subject modified the outcome, with “positive remarks” and “signs of affection” (parental warmth), respectively, leading to a reduction in negative symptoms and improved social functioning (24). Conversely, one study found that family therapy improved positive symptoms, but not negative symptoms or social functioning (25). Criticisms from family members are linked to high levels of cortisol, a marker of stress, in patients and especially in UHR patients (26). Finally, a study concerning extended social functioning also showed that the low quantity or quality of social relations was associated with greater severity of symptoms which classify subjects at risk, within the general population, and within the UHR subgroup (27).

In this study, we were interested in the relationship between the family environment and the risk of PT. Previous research has shown that a quality social support network, and in particular family support, can be a protective factor against the risk of PT, as it is for the suicide crisis (28), in association with an individual factor, the *coping* (29).

The study aimed to assess whether the quality of family functioning is associated with PT in UHR young people aged between 11 and 25 years after a year-round of follow-up.

## 2. Methods

### 2.1. Study design and population

Forty-five patients aged between 12 and 25 years consecutively consulting at the emergency unit of the Louis Mourier hospital in Colombes, France, as part of the Adolescent Psychiatry System were included from January to November 2017. The main reasons for consultation were suicidal ideation (49%), suicide attempts (31%), and self-inflicted scars (31%). Inclusion criteria for the study were being between 11 and 25 years of age, seeking help, and being willing to complete a full questionnaire. Exclusion criteria were not speaking French well-enough or not having the agreement of the adolescent or his/her parents. Follow-up assessments were conducted 8–14 months later.

### 2.2. Baseline data collection

At baseline, sociodemographic data were collected, including age, gender, presence of previous or current psychiatric follow-up, family medical and psychiatric history, psychopharmacological treatment at admission and discharge, substance use disorder, and suicidal history.

The initial assessment was made by a trained psychiatrist. It included an exploration of attenuated psychotic symptoms (APS) with the CAARMS (which is validated for subjects over 14 years of age and whose validity we extrapolate for subjects between 12 and 14 years), depressive symptoms with the Child Depression Inventory (CDI) and the Hamilton Depression Rating Scale (HDRS-21), suicidal thoughts with the Scale for Suicidal Ideation (SSI), negative symptoms with the Self-evaluation of Negative Symptoms (SNS). All scales are validated in their French versions (5, 30). The quality of intra-family relationships was assessed using the Global Functioning (GF) subscale of the McMaster Family Assessment Device (FAD). The FAD is a self-administered questionnaire developed in 1983 (31) and validated in its French version (32) that explores the different dimensions of McMaster's family functioning model (33) through 60 items, divided into six subscales (problem-solving, communication, roles, affective expression, affective investment, and behavior control). The FAD also includes a seventh subscale, the FAD-GF, which is the one used in this study, that assesses global family functioning. It consists of 12 items in which patients indicate their level of agreement with each statement using a 4-point Likert scale ranging from “strongly agree” to “strongly disagree.” Scores are assigned to each response and averaged over the 12 items, resulting in a total score that can range from 1 to 4, with higher scores indicating greater dysfunction (34). Other symptoms were assessed at inclusion with validated French versions of the following scales: the Pittsburgh Sleep Quality Index (PSQI) (35), the Game Addiction Scale (GAS) (36), the Cannabis Use Disorders Identification Test (CUDIT) (37), and the Eating Attitudes Test (EAT-26) (38).

### 2.3. Attenuated psychotic symptoms detection

The use of the CAARMS scale allows for the clinical assessment of patients and the classification of them into non-at-risk subjects (NAR), psychotic subjects, vulnerable subjects, attenuated psychotic subjects, and subjects who have had a spontaneously resolving acute psychotic episodes (Brief Limited Intermittent Psychotic Symptom (BLIPS) group). This classification is based on a set of symptoms presented over the past year and lasting for < 5 years. A patient is said to be at UHR of PT if he is classified in the vulnerable, attenuated psychosis or in the BLIPS group, and that he fulfills two other necessary conditions: being a care seeker (“help seeker”) and having an altered overall functioning (a drop of more than 30% on the Global Assessment of Functioning scale (GAF) (39) than pre-morbid functioning or < 50% in absolute value, for < 5 years and persisting for at least 1 month in the last year). The scale was administered by a total of two trained psychiatrists, and each patient was re-assessed by the same psychiatrist.

### 2.4. Reassessment and psychotic transition

At the follow-up assessment, data from patients and their families were collected regarding ongoing treatment, changes in psychopharmacological medication, hospitalizations, and significant events that had occurred since inclusion, such as changes in the patient's home or school environment, behavior, or drug use. If this had not been done by the referring physician at the time of the reassessment, the patients were contacted again for an interview to perform new CAARMS and HDRS-21 assessments, by the same psychiatrist as at the initial assessment. If patients did not agree to a clinical follow-up interview, data were collected by telephone. Patients were considered to have transitioned to psychosis if they received a diagnosis of psychosis at the end of the follow-up interview or if their referring psychiatrist confirmed the PT.

### 2.5. Statistical analysis

Patient's characteristics at baseline and follow-up were presented as mean (standard deviation, SD) or median (interquartile range, IQR) for continuous variables, and as a number (percentage) for categorical variables. *T*-tests for continuous variables and chi-squared tests for categorical variables were used to test for differences.

Kaplan–Meier curves and Cox regressions were used to analyze survival data, with psychotic transition as the outcome and results were expressed as hazard ratios (HRs) and a 95 % CI.

A univariate Cox regression analysis was performed with baseline characteristics of patients (*p* < 0.1). A dual-direction stepwise procedure based on the Akaike information criterion statistic was performed for variable inclusion in the multivariate Cox regression analysis. All variables included in the final model were assessed for interactions. Cox Proportional Hazard assumptions were tested based on the scaled Schoenfeld residuals.

All tests were two-tailed and a *p*-value < 0.05 was considered statistically significant. All analyses were performed using R (version 4.2.2).

The use of medical data from the electronic health record for research was authorized by the Committee on the Evaluation of Ethics of Biomedical Research Projects of Paris Nord Hospitals (authorization CEERB N 2019-032).

## 3. Results

### 3.1. Population characteristics and PT

The characteristics of the patients at baseline and reassessment are presented in Table 1. At inclusion, 26 (57.8%) of 45 patients were classified as UHR by the CAARMS, and 19 patients (42.2%) were classified as NAR. Of the 45 patients included, 8 were lost to follow-up or refused a new interview, 13 were reviewed for a clinical interview, and 24 were contacted by telephone. The reassessed patients, as well as their parents or foster family and the various caregivers, primarily their treating psychiatrist, responded to a questionnaire.

**Table 1:** Characteristics of patients.

	**Total (*n* = 45)**	**NAR (*n* = 19)**	**UHR (*n* = 26)**	***p***
**Age**	14.906	14.923	14.893	0.97
[11,15]	24 (53.3)	9 (47.4)	15 (57.7)	
[16,20]	19 (42.2)	10 (52.6)	9 (34.6)	
[21,25]	2 (4.4)	0 (0)	2 (7.7)	
**Female gender**	33 (73.3)	18 (94.7)	15 (57.7)	0.006
**Psychiatric follow-up**				0.05
Previous	15 (33.3)	10 (52.6)	5 (19.2)	
Current	20 (44.4)	5 (26.3)	15 (57.7)	
None	10 (22.2)	4 (21.1)	6 (23.1)	
**Family psychiatric history**	22 (48.9)	10 (52.6)	12 (46.2)	0.67
**Psychopharmacological treatment at baseline**	18 (48.6)	5 (31.2)	13 (61.9)	0.06
Antidepressant	5 (11.1)	0 (0)	5 (19.2)	
Antihistaminic	2 (4.4)	1 (5.3)	1 (3.8)	
Antipsychotic	2 (4.4)	0 (0.0)	2 (7.7)	
**Psychopharmacological treatment at follow-up**	9 (20.0)	1 (5.3)	8 (30.8)	0.03
Antidepressant	8 (21.6)	3 (18.8)	5 (23.8)	
Antihistaminic	2 (5.4)	2 (12.5)	0 (0)	
Lithium	2 (5.4)	0 (0)	2 (9.5)	
Antipsychotic	7 (18.9)	1 (6.2)	6 (28.6)	
**Active substance use disorder**	4 (8.9)	1 (5.3)	3 (11.5)	0.46
**Previous suicidal attempt**	15 (33.3)	4 (21.1)	11 (42.3)	0.13
**HDRS-21 score**	13.689	10.789	15.808	< 0.001
**FAD-60 score**	2.476	2.382	2.545	0.36
[0–2.2]	17 (37.8)	7 (36.8)	10 (38.5)	
[2.2–2.6]	17 (37.8)	8 (42.1)	9 (34.6)	
[2.6–2.8]	11 (24.4)	4 (21.1)	7 (26.9)	
**Family structure**				0.38
With two parents	22 (48.9)	7 (36.8)	15 (57.7)	
With single parent	21 (46.7)	11 (57.9)	10 (38.5)	
In institution or foster	2 (4.4)	1 (5.3)	1 (3.8)	
**Psychotherapy**				0.28
Supportive	23 (62.2)	11 (68.8)	12 (57.1)	
Cognitive-behavioral	10 (27.0)	4 (25.0)	6 (28.6)	
Psychodynamic	3 (8.1)	0 (0.0)	3 (14.3)	
Psychoeducation	1 (2.7)	1 (6.2)	0 (0)	
**Reassessment method**				0.39
Clinical interview	12 (32.4%)	4 (25.0%)	8 (38.1%)	
Telephone interview	25 (67.6%)	12 (75.0%)	13 (61.9%)	
**Hospitalization between inclusion and reassessment**	10 (27.0)	2 (12.5)	8 (38.1)	0.08
**Family event between inclusion and reassessment**				0.273
Parental separation	2 (5.3)	2 (12.5)	0 (0)	
Birth of a sibling	1 (2.6)	1 (6.2)	0 (0)	
Departure of an elder sibling	1 (2.6)	0 (0)	1 (4.5)	
House move	3 (7.9)	2 (12.5)	1 (4.5)	
Death of a family member of 1st or 2nd degree	4 (10.5)	1 (6.2)	3 (13.6)	
**Scholar event between inclusion and reassessment**				0.644
Scholar decline	6 (13.3)	3 (15.8)	3 (11.5)	
Repetition	1 (2.2)	0 (0)	1 (3.8)	
Interruption of studies	8 (17.8)	2 (10.5)	6 (23.1)	
Change of institution	7 (15.6)	4 (21.1)	3 (11.5)	

Characteristics include the classification of patients as UHR of PT or NAR, according to the APS assessed by the CAARMS scale. The age range is expressed in average and standard deviation (SD). UHR, ultra-high risk; PT, psychotic transition; NAR, not at risk; APS, attenuated psychotic symptoms; CAARMS, Comprehensive Assessment of At-Risk Mental States; HDRS-21, Hamilton Depression Rating Scale; FAD-GF, Family Assessment Device—Global Functioning.

**Figure 1:**
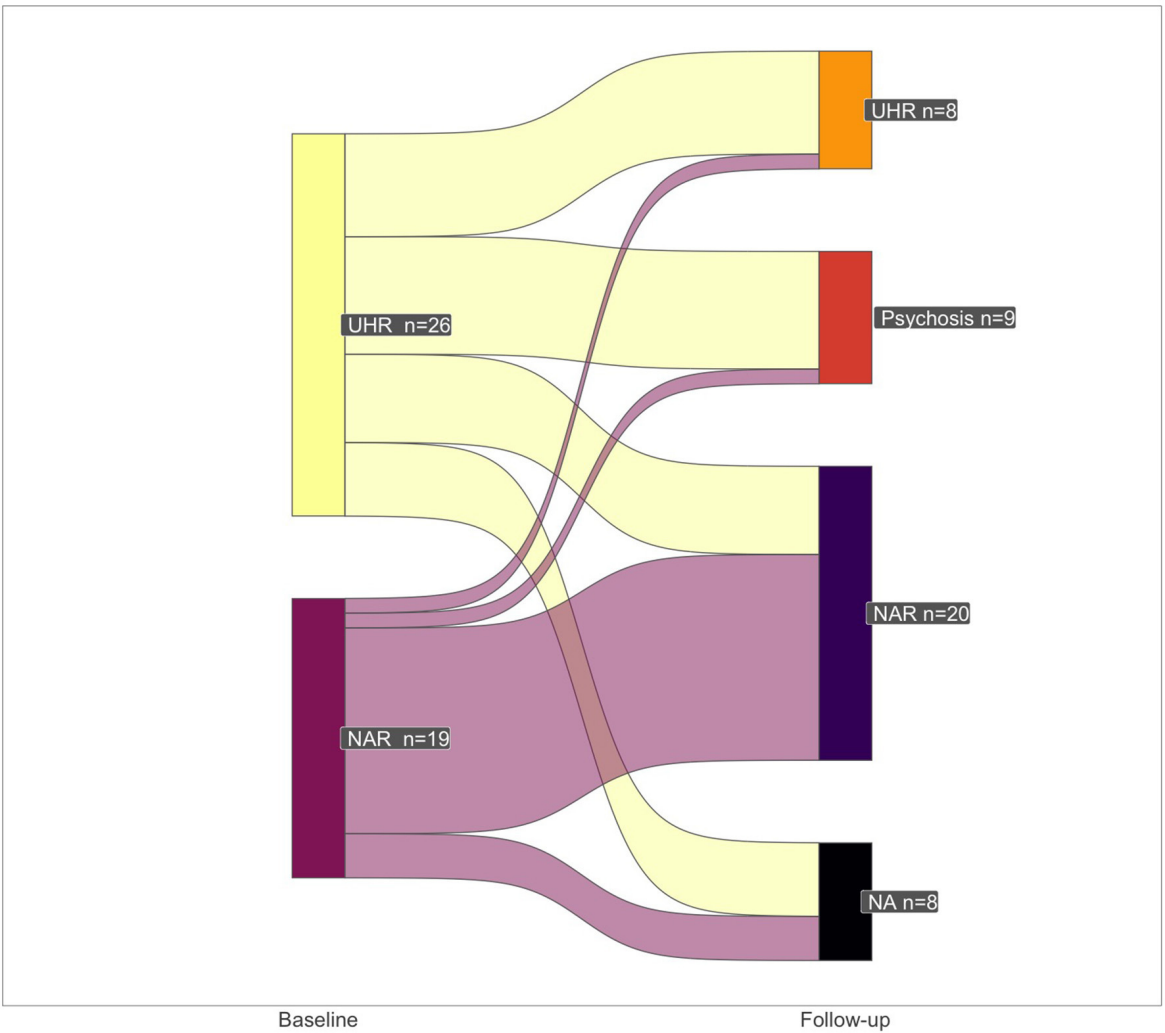
Flow diagram for CAARMS-assessed psychosis status assessed by the CAARMS between inclusion and reassessment. Patients were classified as UHR or NAR at the initial assessment. The chart shows the number of patients who transitioned between psychosis, UHR status, or NAR based on their initial assessment. UHR, Ultra-high risk; PT, psychotic transition; NAR, not at risk; NA, lost of sight; CAARMS, Comprehensive Assessment of At-Risk Mental States.

Figure 1 presents the evolution of the CAARMS evaluation between baseline and reassessment. Of the 37 patients reassessed at 8–14 months, 9 (24.3%) had transitioned to psychosis, and 7 (18.9%) were still classified as UHR and had not transitioned. Out of the 17 patients classed as psychotic or UHR at reassessment, 2 were classified as NAR at inclusion. Six patients classified as UHR at inclusion were classified as NAR at the time of reassessment.

Thirteen patients (35%) had not initiated or discontinued their psychiatric or psychological follow-up. Among these patients, only one (7.7%) transitioned to psychosis, while eight (33.3%) in the group of patients who continued their follow-up transitioned to psychosis (*p* = 0.09). Ten patients (27%) were re-hospitalized at least once during the study period, with two of these hospitalizations being related to treatment interruptions. During the study period, 15 patients experienced a significant family event, including parental separation (*N* = 2), the birth of a sibling (*N* = 1), the departure of an older sibling (*N* = 3), house move (*N* = 5), death of a family member (*N* = 3), and placement in a foster family (*N* = 4). In terms of academic status, 10 (27%) patients interrupted their studies, 8 (21.6%) experienced a decline in academic performance, with 1 patient repeating a year, and 13 (35.1%) changed institutions. No significant difference was found according to the information collection procedure (clinical follow-up interview or telephone).

Regarding depressive symptoms, the mean HDRS-21 over all patients was 13.7 (SD 5.1) at baseline, and 9.2 (SD 8.2) at the time of reassessment (*p* = 0.005). Baseline HDRS-21 was higher in the dysfunctional family group (FAD-GF > 2.2) than the non-dysfunctional family group, but not significantly (*p* = 0.08): 14.7 (SD 5, 6) and 12.0 (SD 3.9), respectively. The HDRS-21 at reassessment was higher, although not significantly (*p* = 0.29) between the patients who interrupted their follow-up and those who continued it: 11.4 (SD 10.4) vs. 7.9 (SD 6.7).

### 3.2. Survival analysis

Table 2 shows results from univariable Cox regression analysis. The initial depression score assessed with the HDRS scale is associated with PT (HR = 4.79, *p* = 0.03), as well as the EAT-26 score at baseline (HR = 1.06, *p* = 0.01). Other variables were not associated with the univariable model.

**Table 2:** Results from univariable Cox regressions for psychotic transition prediction, within 14 months of follow-up.

	**HR [IC 95%]**	***p***
**Age**	1.24 [0.95–1.62]	0.12
**Male gender**	0.73 [0.15–3.66]	0.7
**Scholarly level**		
Elementary school	Ref	-
College and high school	0.35 [0.06–2.16]	0.26
**Previous psychiatric follow-up**	0.54 [0.13–2.15]	0.38
**Family psychiatric history**	2.44 [0.58–10.23]	0.22
**SSI score**	1.01 [0.92–1.1]	0.9
**CAARMS score**	4.36 [0.54–35.5]	0.17
**FAD-GF score**		
[0–2.2]	3.84 [0.71–20.63]	0.12
[2.2–2.6]	Ref	-
[2.6–2.8]	0.77 [0.07–8.52]	0.83
**HDRS-21 score** **>** **18**	4.79 [1.19–19.31]	**0.03**
**CDI score**	1.01 [0.94–1.09]	0.79
**GAS score**	0.97 [0.92–1.01]	0.11
**SNS score**	1.02 [0.97–1.08]	0.41
**CUDIT score**	2.52 [0.3–21.51]	0.4
**PSQI score**	0.9 [0.77–1.04]	0.15
**EAT-26 score**	1.06 [1.01–1.1]	**0.01**

HR, hazard ratio; [IC 95%], confidence interval at 95%; SSI, Scale for Suicidal Ideation; CAARMS, Comprehensive Assessment of At-Risk Mental States; FAD-GF, Family Assessment Device—Global Functioning; HDRS-21, Hamilton Depression Rating Scale; CDI, Child Depression Inventory; GAS, Game Addiction Scale; SNS, Self-evaluation of Negative Symptoms; PSQI, Pittsburgh Sleep Quality Index; CUDIT, Cannabis Use Disorders Identification Test; EAT-26, Eating Attitudes Test; HRDS score was considered as a categorial variable to assess severe forms of depression. Values in bold are the names of categorical variables.

Table 3 shows results from multivariable Cox regression analysis. Age, UHR status at baseline, and the lowest level of FAD-GF score were associated with PT, with an HR of, respectively, 1.81, 11.18, and 0.09, *p* < 0.05. The confidence intervals for our estimates are wide due to the limited sample size and the low number of events observed. EAT-26 sore was not significantly associated with the psychotic transition in the multivariable model and was, therefore, not included. The selected model presented high statistical properties, with a concordance rate of over 0.85.

**Table 3:** Results from multivariable Cox regression for psychotic transition, within 14 months of follow-up.

	**HR [IC 95%]**	***p***
**Age**	1.44 [1.05–1.97]	**< 0.05**
**UHR at baseline**	11.18 [1.1–113.67]	**< 0.05**
**FAD-GF score**		
< 2.2	0.09 [0.01–0.78]	**< 0.05**
[2.2–2.6]	Ref	-
>2.6	0.11 [0.01–1.03]	0.1

Significant variables in univariate analysis were used for a stepwise procedure selection. HR, hazard ratio, [IC 95%], confidence interval at 95%, UHR, ultra-high risk, assessed with CAARMS scale, FAD-GF, Family Assessment Device—Global Functioning, HDRS-21, Hamilton Depression Rating Scale. Values in bold are the names of categorical variables.

## 4. Discussion

The multivariable survival analysis revealed that better family functioning is a protective factor for year-round PT in UHR subjects and underlines the importance of family interactions. Other expected risk factors, already well-established in previous studies were the age (40, 41) and the UHR status at baseline evaluated by the CAARMS. Given the asymmetrical distribution of the FAD-GF score, and to respect Cox proportional Hazard assumptions, the score was converted into a 3-class factor of equal size. The intermediate level of the FAD-GF score was selected as the reference for analysis due to the limited number of events within patients with high scores and to evaluate the potential protective effect of a non-dysfunctional family. In our population, patients reported high scores on the FAD-GF scale (median score of 2.4 and standard deviation of 0.32). The threshold for this scale to define a family dysfunction is 2.0. (42). Nearly all the study participants (98%) had a score >2 on the questionnaire, with approximately 20% exhibiting particularly high scores. This is explained by the fact that these are patients seeking care, with other studies showing that these patients have higher scores (43). Given that all scores were positive on this scale, we tested several significance thresholds to divide the patients and chose to divide them into three equal-sized groups. No interactions were identified that could explain the lack of a statistically significant difference for the highest class of FAD-GF scores.

Among the 21 patients classified as UHR for psychosis who were followed up, 8 (38.1%) transitioned to psychosis at 12 months. This rate is high relative to most clinical samples (44), which can be explained in part by the emergency help-seeking context in which patients consult. The rate of psychotic transition seems to be higher with the severity of the initial motive for consultation (45–48). Of these UHR patients, 62% had sought care for psychiatric symptoms before inclusion in the study, and 20% were already receiving psychopharmacological treatment. The only notable difference in this population was the low prevalence of substance use, with only three individuals reporting tobacco use and one reporting daily cannabis use. No other chronic intoxications were reported.

Our findings support previous research suggesting that the quality of family functioning is related to improvements in depressive symptoms (23). Within our patients, better family functioning was related to lower levels of both depression and APS. However, for depressive symptoms, this difference did not reach statistical significance (*p* = 0.08).

This study has several limitations. The first is the small sample size, with some lost of sight (*N* = 8). The second is that a large proportion of patients were re-assessed by phone while the CAARMS is initially designed for a face-to-face interview. Additionally, the use of the CAARMS scale to assess PT risk has its limitations. While the scale has a high negative predictive value, with only two out of the patients classified as NAR at inclusion transitioning to psychosis or being reclassified as UHR, the positive predictive value is lower, with only 40% of patients in the UHR group transitioning to psychosis. Moreover, for being considered as UHR, patients must seek care, making it difficult to extrapolate results for the general population. However, there is probably already *a priori*, for this type of patient, a change in attitude and family relationships, compared to the general population who do not seek care. Patients' evolution in terms of education shows, for example, a significant difference with the general population, with 27% of patients having interrupted their education. However, the interest of such a study remains whether a family intervention might be useful in reducing the number of PTs in patients at risk. This, therefore, does not concern patients who do not seek care. The fact that CAARMS is limited to the population of healthcare seekers, therefore, remains interesting in our study, for therapeutic rather than epidemiological purposes.

The FAD-GF used to assess family functioning was a self-administered questionnaire and might be sensitive to conscious and unconscious biases. However, the FAD-GF has been shown to have high sensitivity and specificity in evaluating family functioning and has been widely used in previous research (42). It would have been beneficial to include the perceptions of parents and siblings in the assessment of family functioning to provide a more comprehensive understanding of family interactions. Nevertheless, Byles et al. (49) showed that the score obtained on the FAD-GF scale was correlated with other measures related to family dysfunction, such as alcoholism, “nervous” diseases, legal problems, domestic violence, and separations but was not correlated with factors unrelated to family dysfunction, such as physical illness or geographic location. This supports the validity of the FAD-GF scale for the assessment of global family dysfunction. It would thus have been interesting to administer the FAD-GF at the time of reassessment to assess the impact of psychiatric follow-up, which could potentially modify the overall functioning of the family. However, due to the self-administered nature of the FAD-GF and the fact that some patients were only contacted by phone, it was not possible to administer the scale under controlled conditions.

To the best of our knowledge, this is the first study showing with longitudinal data that better family functioning is a protective factor for PT. This finding supports the idea that improving family functioning may have a positive impact in terms of symptomatic evolution (50), and that early family intervention, in dysfunctional families, could prevent PT in UHR subjects (24, 25, 51–53).

## Data availability statement

The raw data supporting the conclusions of this article will be made available by the authors, without undue reservation.

## Ethics statement

The studies involving human participants were reviewed and approved by Committee on the Evaluation of Ethics of Biomedical Research Projects of Paris Nord Hospitals. Written informed consent to participate in this study was provided by the participants' legal guardian/next of kin.

## Author contributions

JL, VA, CD, and JB contributed to the study design, protocol development, protocol implementation, and data management. VA led manuscript development (literature search, planning, and writing of the first draft). TD performed data analysis and assisted in manuscript development and editing. All authors reviewed the manuscript and approved the submitted version.
